# The realities and expectations of community involvement in COVID-19 research: a Consumer Reference Group perspective

**DOI:** 10.1186/s40900-022-00389-z

**Published:** 2022-09-28

**Authors:** Claire Adams, Paul Albert, Tim Benson, Anne Cordingley, Barbara Daniels, Noreen Fynn, Mary Gurgone, Chris Jeffery, Ann White, Natalie Strobel

**Affiliations:** 1 Kurongkurl Katitjin, Edith Cowan University, 2 Bradford Street, Mount Lawley, Perth, 6050 Australia; 2 School of Arts and Humanities, Edith Cowan University, Perth, Australia; 3 Consumer Reference Group Member, Perth, Australia; 4 Council on the Ageing, Perth, Australia

**Keywords:** Consumer involvement, Consumer perspectives, COVID-19, Health services research, Older adults

## Abstract

**Background:**

Older adults have been disproportionately impacted by the COVID-19 pandemic. COVID-19 restrictions such as stay at home orders and physical distancing measures have been implemented to reduce older adults’ risk of infection, however, such measures can have negative effects on older adults’ mental health and social wellbeing. In 2020, the research team received funding as part of an Australian COVID-19 research grants program to investigate how services can better meet the mental health and social support needs of older adults during COVID-19. A Consumer Reference Group (CRG) was established to provide a community perspective on all research activities.

**Main body:**

The CRG comprised of eight older adults aged 65 years and older living in Western Australia. Two members of the CRG were involved in the initial grant proposal, and one member worked for a not-for-profit organisation that provides support and advocacy for older adults. The CRGs role was to provide consumer and community perspectives on the research design, advise on study materials, facilitate links between consumers, the community, and researchers, and advocate on behalf of consumers and the community. The CRG was encouraged to reflect on the research project, their contributions, and the outcomes obtained. In this commentary, we document the CRGs contributions to the project, and record their reflections, including what went well, what were some challenges, the realities of conducting research during COVID-19, and lessons learnt.

**Conclusion:**

The CRG were active participants in the research process. They shared their perspectives and made important contributions to the project. Through collaboration with the CRG, we were able to reach four key messages, underpinned by consumers lived experiences, that were used to co-develop knowledge translation products. These were disseminated to service providers and older adults.

**Supplementary Information:**

The online version contains supplementary material available at 10.1186/s40900-022-00389-z.

## Plain English summary

Since the start of the COVID-19 pandemic, health and social measures have been introduced to reduce the spread of the virus, including lockdowns, physical distancing, and mask mandates. Older adults (aged 60 years and older) are considered particularly vulnerable to COVID-19 and have therefore faced some of the greatest restrictions to reduce their risk of infection. These restrictions can have a negative effect on older adults social and emotional wellbeing. In 2020 the research team received funding to investigate how services could better meet the mental health and social support needs of older Australians during the pandemic. To enable a community perspective on all research activities, a Consumer Reference Group (CRG) of eight older adults living in Western Australia was established. Two of the eight CRG members were involved in the initial grant proposal. The CRG’s role was to share their thoughts on the research design, study materials, and to provide links to and advocate for consumers and the community. This commentary reports reflections from the CRC on what went well, what some of the challenges were, the realities of conducting this research during COVID-19, and what lessons were learnt. Through collaboration with the CRG key messages for the research project were reached and used to inform infographics, which were then disseminated to inform service delivery providers and older adults of the research outcomes.

**Supplementary Information:**

The online version contains supplementary material available at 10.1186/s40900-022-00389-z.

## Background

Older adults (aged > 60 years) have been significantly impacted by the COVID-19 pandemic. The risk of serious illness from COVID-19 increases with age and/or having a chronic medical condition [1, 2]. As a result, older adults and those with underlying health conditions were told to stay at home, avoid social activities, and physically distance themselves from others [3]. These important international public health messages were implemented to ensure the health, wellbeing and safety of older adults was secured, to reduce the number of COVID-19 cases, and to protect over-burdened health-systems. However, with self-isolation comes other health and social issues such as social isolation. Social isolation can lead to a range of well-evidenced health and social issues including loneliness, poor mental health and wellbeing, lack of community connectedness, and increased complications of already existing health problems [4–6]. Indeed, the consequences of social isolation as a result of COVID-19, and the impact on mental health, wellbeing, and loneliness, have already been highlighted as affecting older adults [7, 8].

During COVID-19 many community-based and non-government organisations as well as small local councils were supporting older adults by amending their service delivery and providing support and resources to ensure older people had access to the help they needed [9–12]. However, there were and still are large numbers of older adults who have been isolated from their communities, who have no family to support them, and who were not receiving the information they needed to access services, or worse, were receiving the wrong information [13].

Having mechanisms in place for older adults to access mental and social support during periods of social isolation is important to reduce stress and mitigate the potential longer term negative ramifications of social isolation to older people’s health and wellbeing. Understanding what support older adults accessed during COVID-19, and how services can provide more effective support in the future, is fundamental to making sure older adults receive high quality care, both now and in future periods of self-isolation.

Patient and public engagement in health services research has been recognised as important to improving researchers understanding of community needs, improving trust and confidence in the research findings, building relationships with communities and improving translation of research outcomes [14, 15]. We consider patient and consumer engagement to be “The active, meaningful, and collaborative interaction between patients and researchers across all stages of the research process, where research decision making is guided by patients’ contributions as partners, recognizing their specific experiences, values, and expertise”. [16]. However, patient engagement is rarely reported in much depth, and there is little evidence regarding the best patient engagement practices [15, 17]. This is particularly the case for older adults, who are rarely the target of patient engagement strategies [18]. Engaging older people as research partners is particularly important as healthcare systems are generally not designed to meet the needs of older people, thus working with patients on research to improve services for older adults can help ensure findings and recommendations meet their preferences and needs [18]. During COVID-19 engaging patients and the public in research has been particularly challenging due to lockdowns and social isolation measures [19]. Nevertheless, the pandemic has highlighted the importance of public cooperation in health care, and the value of public trust in health research methods and findings, which can be fostered through patient engagement [19]. Whilst there is a lack of evidence on the best ways to engage patients and the public in health research, involving a group or team of patients as collaborators on projects, who work with researchers to make decisions on research activities, is the most active approach to patient and public engagement [14]. Indeed, national research funding bodies (e.g., National Health and Medical Research Council in Australia, Canadian Institutes of Health Research) adopt and recommend this approach to ensure patients and the public have a voice in research [20, 21].

The present commentary is a collaboration between two academics and eight members of a consumer reference group (CRG) established in 2020 to advise on a research project entitled ‘Enhancing mental health and social care services for older adults during periods of long-term social isolation’. Our aim for this commentary was for CRG members to reflect on the positives and challenges of being involved in this research during COVID-19 and how CRG members played an important role in the translation of project findings. The commentary was divided into two parts:

- Part A: provides a brief background of the research project including recruitment and role of CRG members to provide context to the participation of CRG members in the research
- Part B: provides reflections of CRG members on their participation in the research.

## Part A

### The present project

**Fig. 1:**
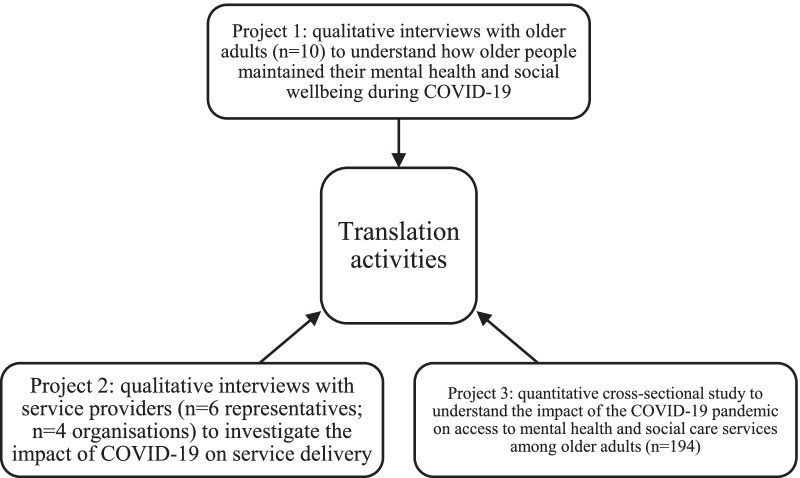
Outline of research project

Throughout 2020–21 in Western Australia (WA), Australia, older adults were encouraged to self-isolate both at the start of the pandemic and during smaller outbreaks to reduce their risk of infection [22]. The period in which Western Australians were most affected by COVID-19 restrictions was from mid-March 2020 to early June 2020. During this time, stay-at-home orders were issued to older adults and social and work restrictions were implemented in the wider community [23]. In 2020, the research team received funding as part of a state-wide COVID-19 research grants program to conduct research investigating how services can better meet the mental health and social support needs of older adults during COVID-19 in WA. Three projects and translation activities were completed to inform the research (Fig. 1). Extensive details of the three projects have not been provided as the purpose of this paper is to report on the involvement of the CRG in the overall program of research. Primary articles of the three projects have been submitted to journals and are currently under review [24–26].

### Consumer involvement: The Consumer Reference Group (CRG)

As part of this research project a Consumer Reference Group (CRG) was established. Two CRG members, CJ and AW, were already engaged in the initial grant proposal. They provided feedback on the proposal and offered strategies for translation activities that were incorporated into the project. When the grant was successful, they became founding members of the CRG, and provided names of potential candidates who they thought may be interested in joining. Other CRG members were recruited through the research team members networks.

The final CRG comprised of eight older adults aged 65 years or older living in Perth, WA. CRG members have between 15 and 40 years’ experience as consumers on research projects. Their experience includes providing consumer representation in research and policy, as members of research teams, managing committees, and advisory committees for universities, government, and not-for-profit organisations. Additionally, one member of the reference group works for a not-for-profit organisation, Council on the Ageing (COTA), which provides support and advocacy for older West Australians. Reference group members were appointed for the length of the project, from September 2020 to mid-2022.

The role of CRG members was to provide a community perspective on all research activities conducted as part of this project. At the initial CRG meeting, members were asked to sign a Terms of Reference document, which outlined their expected contributions. These were:

- Provide consumer and community perspectives around the research design and what should/should not be included in the study’s online questionnaire and qualitative interviews
- Advise on the language used in all study documents, including any lay summaries
- Facilitate links between consumers, the community, and researchers at the administering university
- Advocate on behalf of consumers and the community, where appropriate

Initially members were asked to participate in four meetings, although in practice five meetings were held. Each meeting lasted approximately two hours and members were remunerated per hour for their time.

### Consumers contributions to the project

**Fig. 2:**
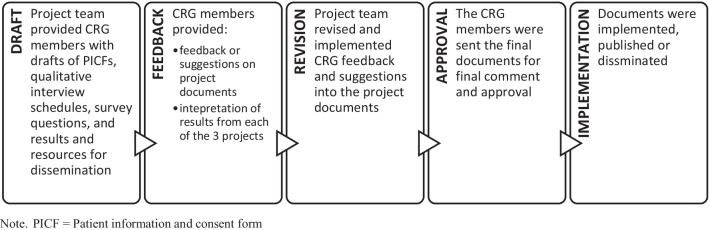
Process for working with CRG members. *PICF *patient information and consent form

The CRG was pivotal to the success of research activities delivered for this project. Through working with CRG members, several changes were made to the project, including edits to key study documents (study information letters and consent forms, interview schedules, survey questions, resources for dissemination), revised interpretations of the study results, and revisions to research manuscripts. Figure 2 outlines the process of working with CRG members and how such changes occurred.

In addition to providing feedback on drafts, the CRG also:

- suggested the use of an abbreviated and full patient information and consent form; participants were provided with both so they could choose which one was easiest for them to complete
- provided names of organisations for participant recruitment for all three projects and translation activities
- forwarded the recruitment communications to their own networks, including individuals and organisations
- suggested communication and consultation strategies to help reach older people who do not have access or who have limited access to electronic communication
- worked collaboratively with the research team at all stages of the project to ensure the research was community focused
- contributed unique insights based on their lived experiences in relation to the projects aims and objectives.

## Part B

### Reflections from the consumer reference group

The following section has been written in first person by members of the CRG (PA; TB; AC; BD; NF; MG; CJ; AW), with support from members of the research team (CA; NS). CRG members met with members of the research team in January 2022 to reflect on the research project, their contributions, and the outcomes obtained. The information below documents CRG members reflections. Authors CA and NS supported members through structuring, phrasing, and adding references to the broader literature, where needed, to provide context to the arguments made.

### CRG reflections: what went well

Our involvement in this project helped to shape the types of questions that were asked of participants, ensuring the research explored issues relevant to older people in the community. For example, we find that loneliness and social isolation among older adults is often overlooked by health and social service providers and think this should be a focus of research, particularly during COVID-19. Through our meetings with the researchers, we were able to raise our concerns and suggest that giving a voice to subgroups who are likely to be at risk of loneliness and mental health declines helps to draw attention to their needs and potentially inform changes to service provision to better meet their needs, which we feel is necessary. By working with the researchers to include questions on loneliness, the project was able to identify a group of older adults who were experiencing social isolation during COVID-19 and needed support yet found it difficult to access services and get the help they require. This makes the results more relevant to the needs older people in the community.

We also find there is a growing reliance on digital technology to gain information and access services, particularly during COVID-19, and think this has had huge implications for the way older people use services. Our involvement in this project encouraged researchers to explore the role of digital technology on service access, which we find can facilitate access to care (e.g., less travel time), but for some older adults can also be a barrier. Many older adults can use technology and are motivated to learn [10, 27], however this research showed that some older people are unwilling or unable to use digital platforms. We think this could be due to low digital literacy, fear of the unknown, or not having up-to-date phones, which are needed to use many online resources. We have already seen that the use of WA’s ServiceWA app (an app to store proof of vaccination status that was required to enter many shops and amenities) was proving difficult, as many older people do not have recent enough phones to download the app. Even though this has repeatedly been raised as a concern by older people’s advocacy groups it is not often seen as a major priority for government, and we think by including questions on digital technology this study was able to explore a prominent issue for older people, which should continue to be attention in research.

We also worked with researchers to help interpret the results. For example, the project found 38.7% of participants had two or less people they felt able to chat with. As a group, we felt this showed that many older adults lack social support in the community, and we think there is a need to increase opportunities for community engagement to foster social wellbeing. This may help to protect against loneliness, irrespective of COVID-19. People who did access social support groups during COVID-19 restrictions reported “feeling better”, which we think demonstrates the importance of engaging with local community groups to facilitate activities that may improve older adults’ wellbeing. Thus, we helped to shape the recommendations made from this project through emphasising the role of informal supports such as community groups in helping older adults cope during the pandemic.

We also recommended that older adults need to be provided with alternative ways to access services during the pandemic, such as face-to-face care. This is important to facilitate older adults use of mental health and social support services. Whilst telehealth may increase access to mental health care during COVID-19, for older adults, an overreliance on digital technology may exclude them from being able to access services that meet their psychological and social needs. In addition, issues such as hearing loss, English as a second language and a lack of culturally appropriate services impact older adults’ capacity to access relevant and appropriate supports, and this needs to be considered when planning service delivery.

A major positive to come out of this research, based on the reports and results we read, is that older adults reported enjoying being interviewed and valued sharing their lived experiences with researchers. We know of people who chose not to participate in the research due to stigma of mental illness and concerns their opinions will not be valued, however some people later reflected that they may have benefitted from being interviewed. This means participating in the study may have helped people process their emotions and feel part of their community, which was particularly important during COVID-19, as opportunities for community engagement were more limited. By gaining insight into older adults’ preferences and needs, we as a consumer reference group were able to make recommendations for service provision on behalf of older people in our community that we believe were appropriate and relevant to their needs.

### CRG reflections: What were some challenges

Although we were able to help shape the questions being asked, and recommendations made, participant recruitment was challenging. We provided the research team with avenues of recruitment in an effort to gain a diverse sample. The researchers used these avenues; however, we would like to have seen more participants recruited that live in non-metropolitan areas, who had poor mental health, were of Aboriginal and/or Torres Strait Islander origin and/or were from culturally and linguistically diverse (CALD) backgrounds. It was disappointing to see few people from diverse backgrounds participate. On reflection, it is possible that we did not use our existing networks sufficiently to access a broader sample. After discussions with the research team, we think there could have been more follow-up contacts with organisations and individuals who had expressed an interest in participating or who were yet to respond to our initial contact. Tailoring recruitment communications to attract people of diverse backgrounds may also have assisted with the recruitment of these populations. There was opportunity for the research team to work with us to further explore recruitment options to gain a more diverse sample, however, funding restraints limited the time that could be spent on our collaboration efforts to obtain a broader participant sample.

We were advised that funding for interpreters was not possible, however we think such funding is necessary to enable the inclusion of CALD older adults in the project and should be a priority in future research. We also believe older adults were experiencing ‘survey fatigue’, as based on our experiences there have been many requests for participation in health and wellbeing surveys during COVID-19. This may have adversely impacted the size and breadth of the sample that was obtained. After discussion with the research team, and based on our own experience as consumers in research, we are aware that funding dedicated to COVID-19 research and COVID-19-related publications increased in 2020, both in Australia and overseas [28]. Therefore, the demand for participants in COVID-19-related research was high and this would have likely explained some of the challenges in recruiting participants for the project. Greater funding and longer timelines would allow more time for us to work with the researchers on participant recruitment to ensure the most vulnerable members of the community are not overlooked.

### CRG reflections: realities of conducting research during COVID-19

Conducting research during the pandemic may be a useful way older adults can express their frustrations and process their emotions; however, it may also act to exacerbate concerns. Due to social isolation measures and fears of catching COVID-19, older people appear to be less willing to engage in research, as participants and collaborators. Upon reading participants qualitative responses, it was clear that some older adults enjoyed the peace and quiet of isolation but there was also a lot of anger in the community regarding COVID-19 restrictions and fear of catching COVID-19. We encourage researchers and the wider community to be mindful that COVID-19 is a time of heightened arousal and research participants and collaborators may require additional psychological and physical support to contribute to research projects.

There is also a lot of misinformation regarding COVID-19 in the media, which we have found confusing, and this was manifested in participants responses. This may have resulted in a lack of trust in research and health institutions, which could impact the quality of patient engagement. A lack of clear and consistent messaging related to COVID-19 has been recognised by the World Health Organization, who declared an ‘infodemic’ due to the abundance of false or misleading information [29]. It is important for researchers to be aware that older adults may be hesitant to participate in research during- and post-COVID-19 due to confusion as to its legitimacy and/or benefit to the community.

### CRG reflections: lessons learnt

One of the main lessons learnt from this project is the value consumers can play in shaping the recommendations derived from research. We highlighted the importance of local community organisations in supporting older adults’ mental health and social wellbeing during COVID-19 and think there is an opportunity for community-based organisations to better engage older adults and provide services that facilitate social connectedness to protect against social isolation. These suggestions informed the policy and practice implications from the project. We were also able to shape future research directions, by encouraging the inclusion of active local councils who provide social support services for older adults, to understand what their role is and how they have supported older adults during COVID-19 restrictions. Such information could help to inform recommendations for service delivery in other council areas. We also suggested that community centres, which are often adept at connecting with older adults and vulnerable populations, could have been targeted to gain insight into the services they provide that facilitate social networks. For example, many older people access community centre courses, events, libraries, and resources to gain information and socialise in a safe environment. During COVID-19, courses and events were ceased and/or postponed, and libraries were temporarily closed. Funding for local councils and community centres to provide stronger outreach services, such as mobile libraries and internet services, may have been useful during this time, and warrant further investigation. This was revealed when looking at the study results with researchers and discussing the relevance of the results for older people. We feel consumers have an important role in helping researchers to conduct research of relevance to communities.

### CRG reflections on consumer involvement in this research project

Overall, we enjoyed contributing to this project and hearing each other’s perspectives. Being involved in this research provided us with an opportunity to broaden our understanding of the challenges faced by older people in our community and gain an appreciation of the impact of COVID-19 on people from different socio-economic backgrounds. It also allowed us to reflect on our own experiences during COVID-19, consider how we have coped, and how we can better support others. We were able to inform the types of questions being asked and helped the researchers to reach recommendations for service delivery that meet older adults needs.

We feel there was a genuine attempt to involve consumers in this research through collaboration across all stages of the project and shared decision-making in the research directions taken. We felt that our opinions were respected and valued and that we were working together on a worthwhile project that could benefit the community and generate greater awareness of the needs of older people. As older people, we can often be overlooked and devalued; contributing to this research helped us to feel useful and appreciated for our experience and knowledge. It is reassuring to know that people working with older adults are willing to listen to our opinions. Too often the knowledge and wisdom that older people can contribute to a project is lost. Projects that meaningfully engage aged consumers will always benefit from the collective insights that we can bring.

We feel that our involvement was well organised, and the number of meetings were appropriate. There could have been more diversity in the CRG composition, as most of us were not significantly impacted by COVID-19 lockdowns, and we generally shared the same views. For example, the inclusion of an older person from a regional/remote area may have helped to identify how services performed outside metropolitan areas and highlight the needs of people in these areas. Furthermore, being given access to some of the studies that were mentioned by the researchers and referenced in the project manuscripts may have helped us to improve our contribution to the project, gain a better understanding of the topic, and would have been of interest to read. We hope that our contributions to this project will inform future research and practice, so that service providers are better resourced and better prepared to respond to the needs of older adults in the future.

## Knowledge translation

The CRG was pivotal in shaping the dissemination of knowledge from this study to key stakeholders, including older adults and service providers. Firstly, the CRG came to a consensus on the key messages and recommendations of the project at the final reference group meeting (Table 1). The CRG then worked in partnership with the research team to develop infographics to distribute to study participants, services who provide care to older adults, and older adult advocacy groups. A meeting was arranged at the translation stage of the project with the CRG and research team to decide on the design, layout, and avenues for dissemination. Content from the key messages and recommendations was also used to inform the materials. Two infographics were developed, one to share how older adults coped during COVID-19, and one to service providers experiences in delivering care to older people during the pandemic (Additional file 1). The CRG also drew on their existing relationships with health care organisations and local councils to help share the results through community groups. Thus, working with the CRG to develop research dissemination tools and distribute them to stakeholders greatly improved our knowledge translation efforts and may enhance the uptake of the findings.

**Table 1:** Key messages from the research project driven by the CRG

1. The role of digital technology in improving access to health information and services for older adults has been over-estimated.
2. Clear and consistent messages related to COVID-19 are required to overcome confusion and mistrust.
3. The role of local governments and community organisations in providing mental health and social support for older adults during and post COVID-19 has been underestimated.
4. The importance of understanding and addressing social isolation among at-risk subgroups of older adults (e.g., those living in rural/remote regions, those with limited social networks, and CALD groups) has been overlooked in research and service delivery to date.

## Discussion

In this commentary, we wanted to provide members of the CRG with an opportunity to reflect on the strengths and challenges of conducting research during COVID-19 and disseminate their learnings from being involved in a COVID-19 research project. CRG members felt there were many strengths to this project, including generating greater awareness of the needs of older adults during COVID-19 and identifying subgroups that require greater social support and services. Including a more heterogenous sample with different language and cultural needs was deemed important by the CRG to provide greater insight into the experiences of marginalised groups. Gaining further perspectives of service providers who deliver social support to older adults in the community was also considered imperative to understand what is currently being done, and what improvements can be made, to enhance service delivery for older adults during and after the pandemic. Overall, the CRG reported they enjoyed being involved in the project and felt their opinions were valued by the researchers. The CRG had input into the questions that were asked, helped interpret the results, and highlighted areas for future research and practice. This demonstrates the importance of listening to consumers perspectives to ensure health research meets their expectations and needs. Consumers can provide valuable insight into the relevance of results for their communities and recommend research directions that are of benefit to them.

Members of the CRG were experienced consumers, and thus were already familiarised with research processes and required little to no training to support them in their roles. This meant our CRG was able to advise on decisions made during the design, implementation, and dissemination of the project with relative ease, and make positive contributions to the project. Researchers looking to involve consumers in future studies should be mindful that time and resources dedicated to consumer training and mentoring may be needed to facilitate meaningful consumer involvement [30]. Including a diverse range of experiences, perspectives, and knowledge in a CRG is important to priority setting and translation activities and may facilitate capacity building between researchers and consumers, who learn can from each other’s expertise [31].

We believe that meaningful improvements in service delivery can best be achieved by listening to the voices of the community members they intend to serve. Older adults in the CRG shared the lived experiences of our research participants and were therefore well placed to inform the translation of the research findings into policy and practice recommendations that reflect the needs of the community. Based on the reflections from the CRG described in this article and the researchers experience, key messages of working with a CRG are described in Table 2.

**Table 2:** Key messages for researchers from working with a CRG

1. Ongoing, consistent, and meaningful engagement with consumers across the spectrum of research will result in outcomes that are beneficial to your project e.g., improve relevant knowledge and translation. This can also help to reduce your (researcher) biases and provide alternative perspectives based on lived experience.
2. Ensure that your consumer group has a mix of backgrounds, experience, ethnicity, gender, and ages.
3. Having the same members in the CRG from the beginning to the end of the project is likely to help build strong relationships and trust between members and research staff. This can result in active discussions about the research being proposed.
4. Collaborating and working with the CRG on research and associated materials means that the research is being created and supported by the people for the people it is meant to support.
5. Consumers will use their networks to help share the research, which may provide access to people who may not have previously had the opportunity to participate.
6. Enjoy learning and meeting new people who are passionate about the work you (as a researcher) do and the opportunity to improve on the work you do.

The need to protect older adults against loneliness and social isolation during COVID-19 has been highlighted in the literature; isolation measures can have a negative impact on older adults mental and physical health [32, 33]. Often recommendations to increase older adults access to telehealth and online platforms are made, with a view to improving social connectedness and service use [10, 32, 33]. However, insights from our CRG suggest this may not be the most appropriate approach. Rather than increasing access to telehealth, working with service providers to make online platforms more comfortable and user-friendly may be beneficial. Local governments and community organisations may also be a valuable source of support during- and post-COVID-19. Indeed, there is evidence to suggest community-based organisations can be an important source of psychosocial support for older adults and play a role in delivering services to at-risk groups to reduce social isolation and improve their quality of life [9, 34]. Without the CRG, our project may well have reached different recommendations, such as the provision of greater online resources, consistent with previous research.

## Conclusions

It is clear that the experienced CRG added value to the research project from the outset, helping to frame the research questions, co-develop the study documents, interpret the results, advise on the recommendations made, and disseminate the findings. It is also apparent that CRG members appreciated being consulted and enjoyed being engaged in the research; consumer involvement provided them with an avenue to share their expertise, learn from others, feel valued as older people, and contribute to a project of potential benefit to their communities. We encourage researchers to collaborate with consumers across all stages of their research to improve the relevance of the research and translation of results.

## Supplementary Information

**Additional file 1:** Inforgraphics developed for dissemination.

## Data Availability

Not applicable.
