# Supplementary material for: The realities and expectations of community involvement in COVID-19 research: a Consumer Reference Group perspective
Source: Res Involv Engagem. 2022 Sep 28;8:54. doi: 10.1186/s40900-022-00389-z (PMC9516529; doi:10.1186/s40900-022-00389-z)
Supplement: Supplementary file 1 — Additional file 1: Inforgraphics developed for dissemination. [file 40900_2022_389_MOESM1_ESM.pdf]

Coping during social isolation

Through a survey and interviews, older adults in Western Australia shared how they coped during the pandemic.

* Many older adults were resilient:

63% of the older people we surveyed were only slightly affected by COVID-19.

"Everyone knows everyone down here. [During lockdown], most people rang other people to see that they were okay too, because that's the kind of community that it is… [and] the priest was always available [during lockdown]."

Close community networks were an important source of support.

"I haven't experienced any [anxiety around COVID-19], and I didn't know any from other people. It's just a matter of these things occur, and you have to adapt and move on, that's what older people have had to do in our lives, adapt and move on."

Digital and online technologies (e.g., social media, Zoom) were helpful for some people but not for others

– Helped me keep in touch with family and friends

– Saved travel time

– Can't afford, don't have access, don't trust, and can't use social media and zoom.

* However, sometimes it was really difficult:

Two in five only had two or fewer people to chat with.

Nearly one in five older adults said they chose not to access services during the 2020 COVID-19 lockdown even though they would have liked to.

Barriers to accessing support services include:

– the belief that I 'should be able to cope on my own'

– a preference for face-to-face care

Kurongkurl Katitjin
Edith Cowan University

AUSTRALIA ECU EDITH COWAN UNIVERSITY

Additional resources:

Association for Culturally Appropriate Services
Mobile: 0413 619 748
Web: www.afcas.net
Email: info@afcas.net

Council on the Ageing WA
Phone: 9472 0104 (Tues & Wed)
Web: www.cotawa.org.au
Email: policy@cotawa.org.au

Delivering care during social isolation

During the COVID-19 pandemic, organisations like yours have been taking care of isolated and vulnerable older adults in the community.

* Here's what you said worked:

Training in use of Protective Personal Equipment (PPE) and hygiene measures.

- Providing easy-to-understand information
- Staying in touch through phone calls and postcards.

Networking with other organisations to share ideas.

Self care and checking in on co-workers

Providing new services for clients, such as domestic assistance and shopping and delivery.

Our clients say "We can survive if the dishes are not done or if the floor is dirty, but we cannot survive without talking with anyone for weeks." That's very depressing and that's very sad.

It's more internal than external. We are caring for our community and the businesses and everything else, but also self care and how we can help each other to do more for that.

Updating the organisation's website and social media pages with advice on current restrictions, how to get medication, and useful phone numbers.

* Here's what you said you needed:

More time in advance to help clients get comfortable using technology.

Technology upgrades to help you work from home when needed.

More convenient funding in times of crises to adapt and change services.

Kurongkurl Katitjin
Edith Cowan University

AUSTRALIA ECU EDITH COWAN UNIVERSITY

Additional resources:

Council on the Ageing WA
Phone: 9472 0104 (Tues & Wed)
Web: www.cotawa.org.au
Email: policy@cotawa.org.au

Association for Culturally Appropriate Services
Mobile: 0413 619 748
Web: www.afcas.net
Email: info@afcas.net
